# Altered Expression of *ESR1*, *ESR2*, *PELP1* and *c-SRC* Genes Is Associated with Ovarian Cancer Manifestation

**DOI:** 10.3390/ijms22126216

**Published:** 2021-06-09

**Authors:** Monika Englert-Golon, Mirosław Andrusiewicz, Aleksandra Żbikowska, Małgorzata Chmielewska, Stefan Sajdak, Małgorzata Kotwicka

**Affiliations:** 1 Surgical Gynecology Clinic of the Gynecological and Obstetrics Clinical Hospital, Poznan University of Medical Sciences, Polna 33, 60-535 Poznan, Poland; mgolon@ump.edu.pl (M.E.-G.); ssajdak@ump.edu.pl (S.S.); 2 Chair and Department of Cell Biology, Faculty of Health Sciences, Poznan University of Medical Sciences, Rokietnicka 5D, 60-806 Poznan, Poland; azbikowska@ump.edu.pl (A.Ż.); mchmielewska@ump.edu.pl (M.C.); mkotwic@ump.edu.pl (M.K.)

**Keywords:** ovarian cancer, estrogen receptors (ESR1 and ESR2), proline-, glutamic acid-, and leucine-rich protein 1 (PELP1), proto-oncogene tyrosine-protein kinase c-Src (SRC), estrogen signal transduction coregulators

## Abstract

Ovarian cancer remains the leading cause of death due to gynecologic malignancy. Estrogen-related pathways genes, such as estrogen receptors (ESR1 and ESR2) and their coregulators, proline-, glutamic acid-, and leucine-rich protein 1 (PELP1), and proto-oncogene tyrosine-protein kinase c-Src (SRC) are involved in ovarian cancer induction and development, still they require in-depth study. In our study, tissue samples were obtained from 52 females of Caucasian descent (control group without cancerous evidence (*n* = 27), including noncancerous benign changes (*n* = 15), and the ovarian carcinoma (*n* = 25)). Using quantitative analyses, we investigated ESRs, PELP1, and SRC mRNA expression association with ovarian tumorigenesis. Proteins’ presence and their location were determined by Western blot and immunohistochemistry. Results showed that *PELP1* and *SRC* expression levels were found to differ in tissues of different sample types. The expression patterns were complex and differed in the case of ovarian cancer patients compared to controls. The most robust protein immunoreactivity was observed for PELP1 and the weakest for ESR1. The expression patterns of analyzed genes represent a potentially interesting target in ovarian cancer biology, especially PELP1. This study suggests that specific estrogen-mediated functions in the ovary and ovary-derived cancer might result from different local interactions of estrogen with their receptors and coregulators.

## 1. Introduction

Ovarian cancer is one of the deadliest malignant tumors affecting woman. The overall incidence of epithelial ovarian cancers varies from 9 to 17 per 100,000 and is highest in high-income countries. Despite recent advances in research and treatment strategies, it is still the leading cause of death from gynecologic malignancies. Epithelial ovarian cancers mostly occur after the age of 50 years, and this incidence rate increases proportionately with age [1]. Over the last two decades, advances in cancer therapy have greatly improved. Thus, further research is needed to understand how different molecular pathways contribute to ovarian cancer tumorigenesis and to potentiate the discovery of potential therapeutic targets. Studies showed that DNA damage response is a crucial hallmark of high-grade epithelial ovarian tumors, leading to a specific interest in *BRCA* genes. Both *BRCA1* and *BRCA2* genes remain two key tumor suppressors involved in the double-strand breaks DNA repair pathway through the homologous recombination. Additionally, poly (ADP-ribose) polymerase inhibition in *BRCA* mutant tumor cells could induce so-called “synthetic lethality”. When homologous recombination deficiency occurs (due to *BRCA1* or *BRCA2* loss), alternative repair pathways leading, e.g., to chromosome deletions, translocations, and subsequent cell death, are involved. Thus, the development of Poly (ADP-ribose) polymerase inhibitors turned out as a successful medical application. Although, based on the heterogeneity of ovarian cancer, novel agents are still needed. From a translational point of view, a clinical issue related to therapy success is identifying reliable ovarian cancer biomarkers [2].

Currently, there are no effective screening methods that reduce ovarian cancer mortality. Studies using CA125 (Carbohydrate antigen), HE4 (Human Epididymis Protein 4), ROMA (Risk of Ovarian Malignancy Algorithm) index, ultrasonography, and pelvic examinations do not have an acceptable level of sensitivity and specificity [3]. In general, the prognosis of epithelial ovarian cancer is independently affected by FIGO stage, histologic type, grade and maximum diameter of residual disease after cytoreductive surgery. Approximately 75% of all epithelial ovarian cancers are FIGO stage III or IV at diagnosis [4].

Induction of ovarian cancer and its biology is associated with estrogen exposure. Moreover, ovarian cancer cells share a number of estrogen-related pathways with other hormone-dependent tumors [5,6,7]. The activity of estrogens affects formation, etiology, and progression of ovarian cancer [8,9]. The biological role of estrogens is mediated via receptors in a genomic and nongenomic manner involving cytosolic kinases such as c-Src [10,11]. A key mediator of estrogen signaling and actions is proline-, glutamic acid-, and leucine-rich protein 1 (PELP1), also known as modulator of nongenomic activity of estrogen receptor (MNAR). This protein acts as a coregulator, which modulates the genomic and nongenomic functions of estrogen receptors (ESR1 and ESR2) [12]. PELP1 expression was shown to be regulated by the estrogens and their receptors. The *PELP1* promoter, with two estrogen-response element half sites, is upregulated by both ESRs [13]. Additionally, PELP1 acts as a scaffolding protein by coupling ESR1 with c-Src kinase (c-Src/Src), which is required for optimal activation of ESR1, thereby leading to activation of the ESR1–Src–MAPK signaling pathway [14] and other pathways. As reported by Samartzis et al., in ovarian clear cell cancer and endometrioid carcinoma, frequent DNA-damage co-occurs with changes in the phosphatidylinositol 3-kinase pathway. These alternations are related to changes in the *ARID1A* gene. Thus, current and future clinical trials might target proliferative pathways (e.g., the PI3K/AKT/mTOR and the YES1/SRC tyrosine kinase pathways) or metabolic alterations [15]. These data reveal that PELP1 mediates the crosstalk between those signal transducing molecules [16,17]. Moreover, PELP1 participates in PI3K initiation [18], STAT3 transactivation [19], and modulates ESR1–Src–ILK1 signaling. Thus, it promotes cytoskeletal rearrangements, motility, and metastasis [16,20]. The role of the PELP1 protein and its joint action with other molecules in physiological and pathological processes, including ovarian malignancies, has been widely discussed in the literature [11,18,21,22]. Functional studies showed PELP1 interacts with members of the Src kinase family including c-Src, stimulating enzymatic activation. Src axis couples ESR1 with PELP1 and depletion of c-Src inhibited the growth of therapy resistant cancers in in vitro models [17,23].

Ovarian cancer xenografts models revealed that knockdown of PELP1 significantly reduced the tumor growth [24]. Since no direct inhibitors of PELP1 are available, an alternative strategy would be to modify *PELP1* upstream or downstream targets. As an example, cyclin-dependent kinase 2 inhibitors preferentially downregulate the expression of *ESR1* and *PELP1* [11,12,25]. Moreover, liganded-progesterone receptor-beta enhances the proliferative responses to estradiol and IGF1 via scaffolding of ESR1-PELP1-IGF1R-containing complexes [11]. PELP1 deregulation can aberrantly activate the rapamycin signaling that is a target for the ESRs [22]. Thus, an imbalance of the ESR1/ESR2 ratio may have significant consequences.

The plasma membrane ESRs seem to involve post-translational modification (such as phosphorylation) of the receptors, followed by the assembly of a protein complex with some membrane-associated proteins. Proteins from the proto-oncogene tyrosine-protein kinase Src family (SRC) are involved in this process. However, ESRs and SRC interaction and activation remain unclear. Dephosphorylation of the SRC seems to determine interaction with ESRs, causing its phosphorylation and translocation; however, activation of the SRC has been ascribed to ESR1 stimulation, thus creating a complicated circle of activations [26]. Hence, single genes’ expression levels and their expression ratios seem important for the pathognomonic insight in ovarian cancer manifestation.

Taking into consideration the role of estrogen-related signaling pathways, in our study we investigated whether the *ESR1*, *ESR2*, *PELP1*, and *SRC* mRNA expression levels, as well as the generated expression ratios, are associated with ovarian tumor manifestation. Using human (1), noncancerous (2) benign and (3) neoplastic ovarian tissue, we aimed to show if altered gene expression could be pathognomonic and contributes to the progression of ovarian pathological changes.

## 2. Results

Comparison of age, BMI, as well as the menopausal status is provided in Table 1. All groups did not differ according to patients’ BMI. Patients from the control group were significantly younger than patients with ovarian cancer (Me = 53y vs. 63y; *p* = 0.044). For the tissues obtained from patients with benign ovarian changes vs. ovarian cancer patients, the former was significantly younger (Me = 51y vs. 63y; multiple comparison p-value *p* = 0.0357). The patients and controls differed in menopausal status. In the 1st and 2nd subgrouping, significantly more females with diagnosed ovarian cancer became menopausal (*p* < 0.05).

### 2.1. ESR1, ESR2 PELP1 and SRC Relative Expression

**Figure 1:**
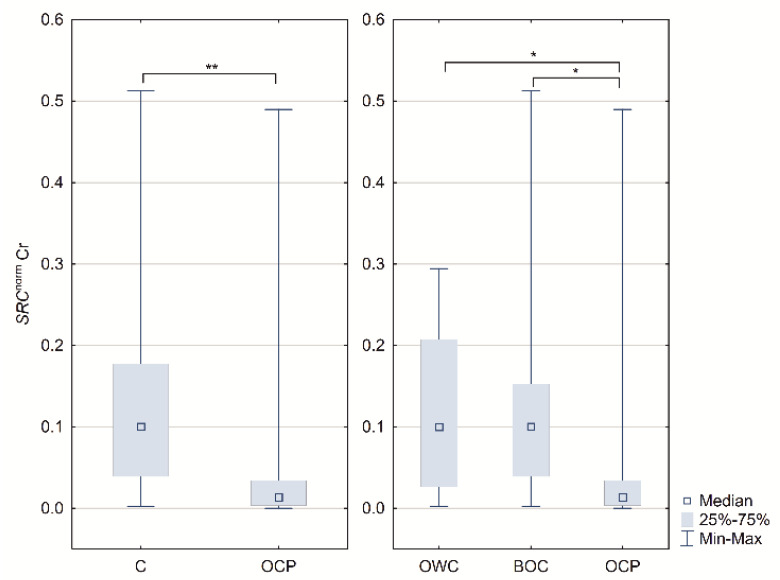
Boxplots of the *SRC* normalized concentration ratios in controls (C), ovarian cancer patients (OCP), ovarian tissue samples lack of any changes patients (OWC) and with benign noncancerous changes (BOC); * *p* < 0.05 and ** *p* < 0.01. Medians, interquartile range and *p*-values are shown in Table 1.

*ESR1*, *ESR2 PELP1* and *SRC* mRNA presence was confirmed in all analyzed samples using specific primers and probes. In case of the 1st subgrouping, the relative expression of *SRC* differs significantly (C vs. OCP; *p* = 0.007). The normalized expression was higher in the control group. In the 2nd subgrouping, the lowest expression was observed in ovarian cancer patients and differed significantly between OWC vs. OCP (*p* = 0.0486), and BOC vs. OCP (*p* = 0.0134) (Table 1 and Figure 1).

**Figure 2:**
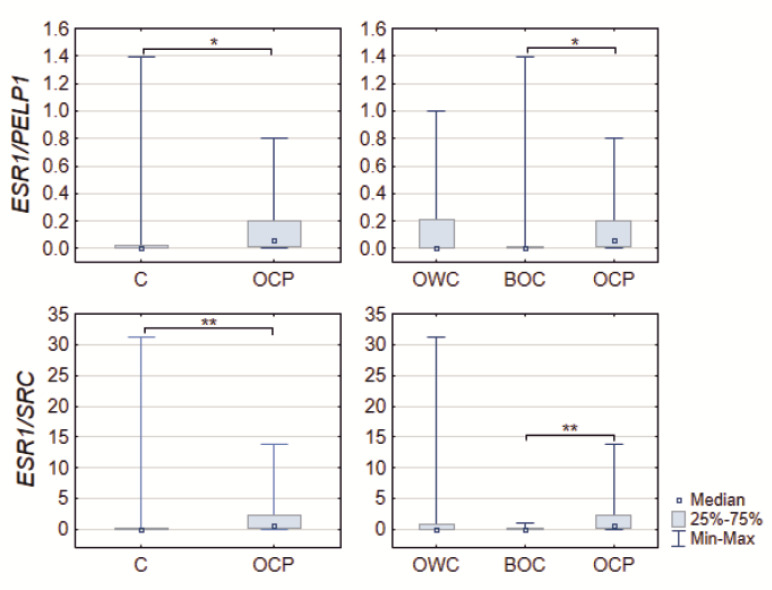
Boxplots of the *ESR1*/*PELP1* and *ESR1*/*SRC* quotient ratios in controls (C), ovarian cancer patients (OCP), ovarian tissue samples lack of any changes patients (OWC) and with benign noncancerous changes (BOC) * *p* < 0.05 and ** *p* < 0.01. Medians, interquartile range and *p*-values are shown in Table 1.

Analyzing the gene expression ratios obtained by from normalized Cr revealed that, in both cases, the *ESR1*/*PELP1* and *ESR1*/*SRC* differed significantly between the analyzed subgroups. First, the controls, in both cases of *ESR1*/*PELP1* as well as *ESR1*/*SRC,* characterized significantly lower ratios (*p* = 0.01898 and 0.00478, respectively). Similar observation was made in case of 2nd subgrouping, but the difference was seen between BOC and OCP patients and not controls. The *ESR1*/*PELP1* and *ESR1*/*SRC* ratios were lower in BOC patients when compared to OCP (multiple comparison *p* = 0.0499 and *p* = 0.006, respectively) (Table 1 and Figure 2).

### 2.2. Relationship between ER Signaling and Ovarian Cancer

Correlation coefficients were compared between groups to determine if a pattern existed related to gene expression changes in ovarian cancer or/and benign noncancerous changes vs. ovarian tissue showing no pathological changes. To avoid data redundancy, we included ovary without changes and benign noncancerous ovary change groups (instead of the control group, which covers both former mentioned). Only data covering correlations regarding normalized expression data and genes’ expression ratios were used.

**Figure 3:**
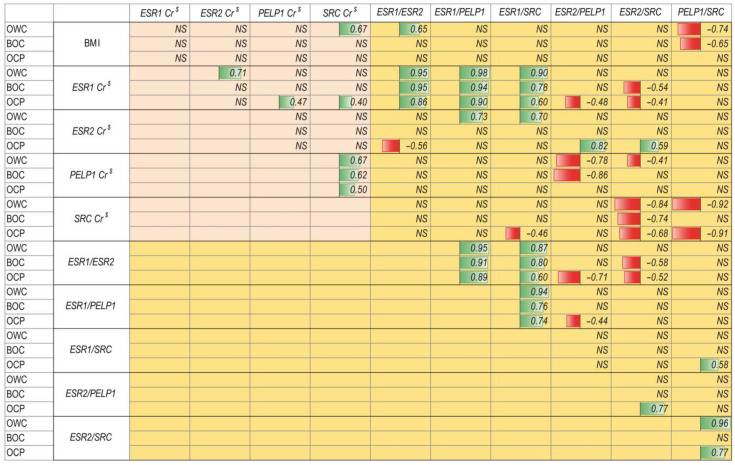
Correlation coefficient plot of ovarian tissue samples lack of any changes patients (OWC) females with benign noncancerous changes in the ovary (BOC) and ovarian cancer patients. Designation: *NS*—not significant; numbers—Spearman rank correlation coefficients; green bars—proportional correlation; red bars—reverse proportional correlation. Green background—clinical data correlations; pink background—gene expression correlations; yellow background—gene expression quotient ratio correlations. Cr ^$^ concentration ratios data normalized using min-max normalization.

In the case of body mass index, a significant strong positive correlation was observed in the OWC group in the case of *SRC* normalized concentration ratio (Cr) data and *ESR1/ESR2* ratio, not observed in both BOC and OCP groups. A negative proportional correlation was observed in the case of BMI and *PELP1*/*SRC* ratio in noncancerous tissue but was not observed in tissue affected by the cancerous process (Figure 3).

Both *ESR1* Cr and *ESR2* Cr correlation showed group-to-group difference in correlation patterns. *ESR1* Cr values strongly positively correlated with *ESR2* Cr only in unchanged tissue and was moderately correlated with both *PELP1* Cr and *SRC* Cr in cancer affected tissue. *ESR1* Cr was very strongly positively correlated with the *ESR1*/*PELP1* and moderately negatively correlated with *ESR2*/*SRC* in both BOC and OCP populations. In case of *ESR2* Cr, cancer affected tissue showed a significant negative correlation with *ESR1*/*ESR2* ratio (moderately strong) and positive with both *ESR2*/*PELP1* (very strong) and *ESR2*/*SRC* (moderately strong). In tissue without changes, *ESR2* Cr strongly positively correlated with *ESR1*/*PELP1* and *ESR1*/*SRC* ratios (Figure 3). *PELP1* Cr was strong and very strongly negatively correlated with *ESR2*/*PELP1* ratios in OWC and BOC groups, respectively, and moderate with *ESR2/SRC* in the OWC group. *SRC* Cr was moderately and negatively correlated with *ESR1*/*SRC* ratios only in tissue affected by cancer. SRC Cr was strong and negatively correlated with *ESR2/SRC* ratio in all groups and with *PELP1*/*SRC* in OWC and OCP groups (Figure 3).

Interestingly, the *ESR1*/*ESR2* ratio was characterized by a strong, negative correlation with *ESR2*/*PELP1* ratio in OCP, and moderately strong with *ESR2*/*SRC* ratio in both benign changes of ovary and cancer affected tissue. In the case of the *ESR1*/*PELP1* ratio, there was only one negative, moderately strong correlation with *ESR2*/*PELP1* ratio in OCP. Only in cancer affected tissue, *ESR1*/*SRC* and *ESR2*/*PELP1* ratios were both positively correlated with *PELP1*/*SRC* (moderately strong) and *ESR2*/*SRC* (strong), respectively. *ESR2*/*SRC* ratio was positively, very strongly correlated with *PELP1*/*SRC* ratio in tissue lack of any changes and strong in cancer affected tissue (Figure 3).

Remaining correlations were not statistically significant or could not be precisely distinguished between the groups. Additionally, some correlations showed decreasing Spearman rank correlation coefficients along with progressiveness of changes (R OWC < R BOC < R OCP) (Figure 3).

**Figure 4:**
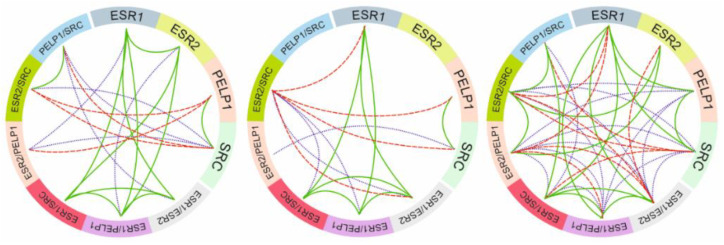
Circular correlation coefficient plot of gene expression and expression quotient. Reverse proportional correlations were indicated by red dash lines, proportional correlation by solid green lines and violet dotted lines indicated significant correlation coefficients relationship in gene-to-gene expression and quotient-to-quotient influence. Ovary without changes (left), benign noncancerous tissue samples (middle), and ovarian cancer (right).

Two-tailed test for correlation coefficients showed different gene-to-gene expression, gene ratio-to-ratio and gene-to-ratio influence and were related to tissue obtained from patients qualified to different groups and physiological/pathological state. As indicated by the violet dotted lines in Figure 4, the pattern varied significantly not only in gene correlation but also in its quotients. In nonchanged tissue, the relationship between genes is uniform and mostly related to correlations described earlier (Figure 3). In benign noncancerous tissue samples, some of the correlations of gene expression and their ratios did not occur. The largest number of connections between the analyzed genes and their quotients were established in the case of cancerous-changed tissue. The *PELP1* and *SRC* expression was significantly linked with other analyzed genes expression level which indicates new network of mutual relationships. However, as shown, altered pathways of expression were also noted in benign noncancerous tissue samples as compared to nonchanged tissue (Figure 4). Additional clinical data correlations and gene expression level correlations were described in supplementary materials and shown in Figure S1.

### 2.3. Protein Expression

**Figure 5:**
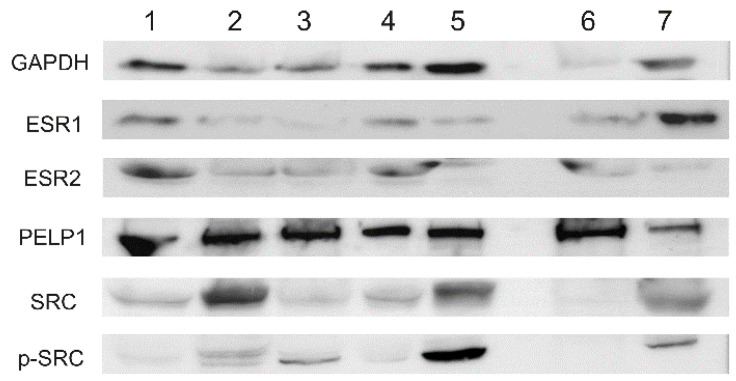
Western blot analysis in selected cases of ovarian cancer tissues (1–5) and noncancerous ovary (6–7).

Western blot analyses confirmed the presence of the analyzed proteins in cancer and noncancer tissue specimens. Immunoreactive bands were observed at the expected sizes (ESR1 66kDa, ESR2 55kDa, PELP1 170kDa and SRC, p-SRC 60kDa). The 37kDa bands of GAPDH reference protein were immunoreactive in all cases (Figure 5). The strongest signal was observed for PELP1 and, in the remaining proteins, the signal was case-dependent and did not show a specific pattern.

**Figure 6:**
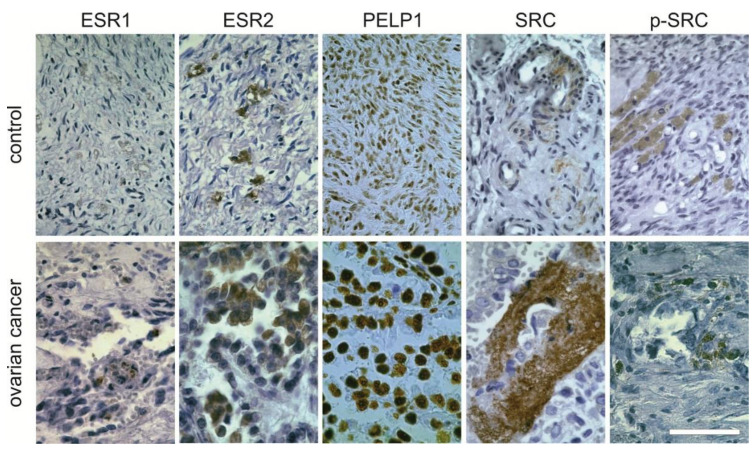
Immunohistochemical staining for analyzed proteins in controls and ovarian cancer tissue. Positive staining is shown in brown. The bar represents 100 µm. This section may be divided by subheadings. It should provide a concise and precise description of the experimental results, their interpretation, as well as the experimental conclusions that can be drawn.

Immunocytochemical staining confirmed the presence of all analyzed proteins. ESR1 and ESR2, as well as SRC, were located in the cytoplasm of the cells, and in the case of unchanged tissue, they were primarily localized in the zona granulosa. On the contrary, the cytoplasmatical staining for p-SRC was observed in the cortical stroma. PELP1 protein staining was positive in the cellular nuclei (Figure 6). The observations in immunohistochemical staining supports those observed by Western blot.

## 3. Discussion

In recent years, attention has focused on genetic factors in cancer development and biology. Abnormal genes expression, activation, and their relationships have been observed in a number of tumors of different origin. In this paper, we explore the expression of four estrogen signaling pathway mediators, *ESR1*, *ESR2*, *PELP1* and *SRC* kinase in ovarian cancer patients.

Despite increasing knowledge in the treatment and etiology of ovarian carcinoma, it remains the leading cause of death due to gynecologic malignancy according to European Cancer Information system (http://ecis.jrc.ec.europa.eu/; last accessed on 16 November 2020). Ovarian cancer is the seventh most common cancer and eighth most frequent cause of death among females, with a five-year survival rate of 45% [27,28]. Age-standardized data show that incidence and mortality rate is the highest in Central and Eastern Europe and slowly decreases in Northern, Southern, to Western European countries. North America ranks slightly higher than Western Europe in incidence and mortality [28]. The risk factors for ovarian cancer include family history, age, menopausal status, as well as the number of childbirths [29]. It has been noted that higher-risk patients have a positive familial history of the disease. In the women of affected first-grade relatives, compared to those with nonaffected relatives, the ovarian cancer incidence is three times more common, but it is also age-dependent [28,30,31]. Overall, testing for moderate- or high-penetrance *BRCA1/2*, *RAD51C*, *RAD51D*, *BRIP1*, and mismatch repair genes for the patients with epithelial ovarian cancer is recommended. Moreover, cascade testing should be offered to relatives of carriers of germline pathogenic variants. Gene sequencing can provide results with different biological meanings. A genetic alteration can be pathogenic or likely pathogenic, benign or likely benign, or finally of uncertain significance. The latter represents the main challenge when interpreting genetic alterations [32]. Our data confirm the higher occurrence of ovarian cancer is age dependent. In the ovarian cancer group, the median age was higher (63 ys) than in the controls (53 ys). A significantly higher occurrence of ovarian cancer was observed in post-menopausal females, which was in line with other authors’ former observations [29,33,34].

Ovarian cancer is considered hormone-responsive cancer with the presence of both estrogen receptors. Their incidence is detectable in 60–100% of the ovarian cancer cases, making them promising therapeutic targets. However, still, selective estrogen receptor modulators have a response rate of 15% in ovarian cancer [5,35]. In our study, all tissue samples were estrogen receptor positive, and their mRNA expression did not significantly differ between samples. It is worth mentioning that we investigated the whole fraction of ESRs and did not distinguish it into different forms. Although the level of mRNA was similar, the correlation of *ESR1* and *ESR2* with other analyzed genes differed. In the case of *ESR1*, in tissue obtained from cancer patients, there was a significant correlation with *PELP1* and *SRC* expression. In addition, this draws attention to the decreasing strength of the *ESR1* correlation, which was the highest in unchanged tissue and the lowest in cancer tissue samples in the case of all analyzed *ESR1* ratios. Additionally, taking into consideration *ESR1-ESR2/PELP1, ESR2/SRC* ratios pathologically changed tissue showed linkage. Similarly, we observed the latter correlations in case of *ESR2*. It suggests the expression level of the gene itself does not determine cancer presence/growth but instead is affected by disturbances in relation to other genes. It could explain the previously mentioned low therapy response, which could be dependent on crosstalk with other proteins/receptors or kinases such as SRC or/and PELP1.

To better understand the role of estrogen receptors in ovarian cancer, we evaluated them together with their coregulators—PELP1 and SRC kinase. PELP1 is the modulator of nongenomic estrogen receptor action and could be the key point of estrogen action mediation via their receptors. In the case of *ESR*s, *PELP1* mRNA expression did not differ in unchanged and ovarian cancer patients. However, both genes’ mRNA levels were higher in the healthy group. It is consistent with the observation described previously by Aust et al. [36] who noticed, in their study, that coexpression of PELP1 and ESR2 proteins was associated with better prognosis for patients with epithelial ovarian cancer. In our study, we revealed with *SRC* that the expression was highly correlated in unchanged tissue and was decreased in ovarian cancer samples. Moreover, *PELP1* expression was strongly related but proportionally reversed to *ESR2*/*PELP1* and *ESR2*/*SRC* expression ratios in noncancerous tissue samples. This observation is supported by other studies that reported a protective effect of PELP1 in cancers [36,37]. Thus, to understand the therapeutic role of estrogen receptors, further studies regarding their coregulators are needed.

Our data shows decreased *SRC* mRNA expression in ovarian cancer patients compared to noncancerous changed and nonchanged. Additionally, the coefficient of variation in the case of nonchanged tissue was the lowest (88%), higher in noncancerous changed samples (112%), and highest in tumor tissues (215%). Our results are in line with the data available in the gene expression profile database (http://ualcan.path.uab.edu; last accessed on 12 December 2020), where the relative *SRC* mRNA level increased with the non-to-pathological stage. This huge variation could influence the results of *SRC* expression and can explain elevated SRC protein levels in ovarian cancer patients observed by others. Taking into consideration the grading and FIGO staging, *SRC* expression was slightly, but not statistically significant, in a higher-grade and poorly differentiated (G3) tumors. However, as described before, production and expression of *SRC* by itself is minimally transforming. On one hand, it was suggested that SRC protein activity played a role in tumor progression. On the other hand, SRC levels played a lesser role in tumorigenesis [38]. This phenomenon can be confirmed by the observation of altered gene expression correlation, especially in tumor tissue. The relation of gene-to-gene and their expression ratios is much more complicated compared to noncancerous samples. Additionally, the SRC protein, in a phosphorylation-dependent manner, interacts with a large number of regulatory pathways in the cell cycle and signal transduction, which influence the carcinogenesis processes in relation to development and cancer progression [39]. Thus, SRC links multiple processes that determine the clinical outcome of a tumor. At the mRNA level, we did not distinguish phospho-SRC from the nonphosphorylated form.

## 4. Materials and Methods

### 4.1. Patients

The study was conducted according to the guidelines of the Declaration of Helsinki and approved by the Institutional Review Board of Poznan University of Medical Sciences (protocol code Nos. 593/19 and 594/19 and date of approval 6/19/2019).

**Figure 7:**
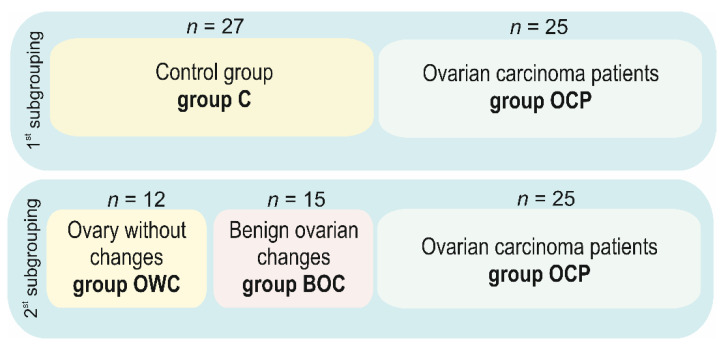
Study material. Diagram showing the number of tissue samples, allocated to different examined groups.

Tissue samples obtained from 52 females (treated at the Surgical Gynecology Clinic of The Gynecological and Obstetrics Clinical Hospital Poznan University of Medical Sciences in 2017–2019) were processed at the Chair and Department of Cell Biology, Poznan University of Medical Sciences. All participants were of Caucasian descent. We used two subgroupings in these studies (Figure 7). The first subgrouping covered noncancerous tissue samples that were obtained from patients who underwent a total hysterectomy (*n* = 27; control group; C) and specimens with confirmed ovarian carcinoma (*n* = 25; ovarian carcinoma patients; OCP). The absence or presence of cancerous changes was confirmed by anatomicopathologic macroscopic and intraoperative microscopic examinations. As shown in Figure 7, in the second subgrouping, the control C group was divided into tissue samples characterized by a lack of pathological alterations (*n* = 12; ovary without changes; OWC) or the tissues where changes were at benign stages and not cancerous in nature (*n* = 15; benign ovarian changes; BOC). The OCP group remained the same.

Histology cancer patient groups were as follows: adenocarcinoma serosum (*n* = 17, FIGO: IC—*n* = 2; IIIA—*n* = 2; IIIB—*n* = 3; IIIC—*n* = 9, IV = 1, G3), undifferentiatum (*n* = 1, FIGO IV, G3) adenocarcinoma clarocellularae (*n* = 1, FIGO IC, G3), adenocarcinoma endometrioides (*n* = 2, FIGO: IIIA G1, IIIB G2), adenocarcinoma mucinosum (*n* = 2, FIGO: IIIB G1), cellulae carcinomatosae (*n* = 1; FIGO IV), foliculoma (*n* = 1).

No patients received chemotherapy or radiotherapy prior the surgery. Samples obtained during surgery were submerged in home-made RNA protective medium [40] (for nucleic acid isolation), preserved in phosphate buffered saline and frozen (for protein extraction), or fixed in 4% paraformaldehyde (for immunohistochemical staining). Tissue samples for RNA and protein isolation were stored at −80 °C.

Median age of cancer patients was 53 yr (interquartile range 55.5–69.5), and the control group was 53 yr (45–67). Taking into consideration the body mass index (BMI), the group consisted of underweight (*n* = 2), normal (*n* = 10), overweight (7), and obese classes I, II, and III (*n* = 3, *n* = 2, and *n* = 1, respectively) carcinoma patients. Among non-ovarian-cancerous patients, BMI showed females of normal weight (*n* = 14), overweight (*n* = 7) and obese classes I, II and III (*n* = 4, *n* = 1, and *n* = 1, respectively).

#### 4.1.1. Clinical Markers Data Evaluation

The CA125 and HE4 markers serum level was determined in all patients. The Roche Elecsys^®^ CA125 II assay is a tumor marker test for use with blood samples to support monitoring and surveillance of ovarian cancer patients, and together with HE4, aids in risk assessment of patients with pelvic mass with the Risk of Ovarian Malignancy Algorithm (ROMA).

Carbohydrate antigen 125 (CA125) and Human Epididymis Protein (HE4) were detected using the full automatic chemiluminescence analyzer Cobas601 along with the corresponding kit and were used according to laboratory protocol (Abbott Laboratories, Roche Diagnostics). Serum HE4 and CA125 levels were calculated for ROMA index value using Roche ROMA index of ovarian cancer risk assessment software. The serum CA125 and HE4 reference ranges were <35 U/mL and <140 pmol/L, respectively.

The ROMA index was calculated according to the levels of CA125, HE4, and menopausal status. Marker values were input to the ovarian cancer risk assessment software, followed by automatic calculation of the corresponding ROMA index. When Roche Elecsys specificity was 75%, premenopausal women with a ROMA value ≥11.4% and post-menopausal women with ROMA value ≥29.9% had a higher risk of ovarian cancer (https://diagnostics.roche.com/global/en/article-listing/roma-calculator.html; last accessed on 16 November 2020).

#### 4.1.2. FIGO Staging and Grading System

Classification rules revised by the Gynecologic Oncology Committee of FIGO are the same for ovarian, fallopian tube, and peritoneum cancer. They are based on findings made mainly through surgical exploration. Operative findings determine the precise histologic diagnosis, stage, and, therefore, patient prognosis.

Epithelial tumors of the ovary are further subclassified by histologic grading, which can be correlated with prognosis. This grading system does not apply to nonepithelial tumors. For nonserous carcinomas (endometrioid and mucinous) grading is identical to that used in the uterus (G1: well differentiated, G2: moderately differentiated, G3: poorly differentiated). Serous carcinomas are graded in a two grade system befitting their biology (high-grade—carry a high frequency of mutations in TP53; low-grade—often contain mutations in BRAF and KRAS and contain wild-type TP53).

#### 4.1.3. Histopatologic Classification

The histopatologic classifications of ovarian, fallopian, and peritoneal neoplasia are as follows: (1) serous tumors, (2) mucinous tumors, (3) endometrioid tumors, (4) clear cell tumors, (5) Brenner tumors, (6) undifferentiated carcinomas, (7) mixed epithelial tumors (composed of two or more of the five major cell types of common epithelial tumors), and (8) cases with high-grade serous carcinoma in which the ovaries and fallopian tubes appear to be incidentally involved and not the primary origin; these can be labeled as peritoneal or serous carcinoma.

### 4.2. Nucleic Acid Extraction and Validation

High molecular weight RNA (HMW-RNA) was extracted from tissue specimens using a double-column system for microRNA and RNA isolation according to the manufacturer’s protocol (A&A Biotechnology, Gdynia, Poland). In short, 20–50 mg of tissue was homogenized using a ceramic pestle and mortar in liquid nitrogen and suspended in 800 μL Fenozol reagent (A&A Biotechnology, Gdansk, Poland). Only the HMW-RNA (without microRNA fraction) was recovered from silica matrix columns for further analysis. The quality, quantity, and purity of HMW-RNA were analyzed as described previously [41] with the use of a NanoPhotometer^®^ NP-80 (IMPLEN, München, Germany). The integrity was evaluated by electrophoretic separation under denaturing conditions [41].

### 4.3. Reverse Transcription and Quantitative PCR

#### 4.3.1. RNA Extraction and Validation

The complementary to RNA DNA (cDNA) was synthesized using a three-step reaction conducted in accordance with Transcriptor Reverse Transcriptase manufacturer’s protocol (Roche, Manheim, Germany) in a total volume of 20 μL. In the first step, a mixture of 5 mM oligo(d)T_10_,1 mM random hexamer primer (Genomed, Warsaw, Poland), HMW-RNA (1 μg), and RNase-, DNase- and pyrogen-free water (Thermo Fisher Scientific, Waltham, MA, USA) was incubated 10 min at 65 °C. Subsequently, the samples were chilled on ice. In the second step, the HMW-RNA mixture was supplemented with 5 U/rx ribonuclease inhibitor (RNasin, Roche, Manheim, Germany), 10 U/rx transcriptor reverse transcriptase (Roche Manheim, Germany), 0.1 U/μL E*. coli* poly(A) polymerase, 0.1 mM adenosine triphosphate (New England BioLabs, Ipswich, MA, USA), 100 mM deoxyribonucleotide triphosphates (Novayzm, Poznan, Poland) and 1× reaction buffer (Roche Manheim, Germany). The subsequent steps of cDNA synthesis were described previously [41].

#### 4.3.2. Real Time Polymerase Chain Reaction

**Figure 8:**
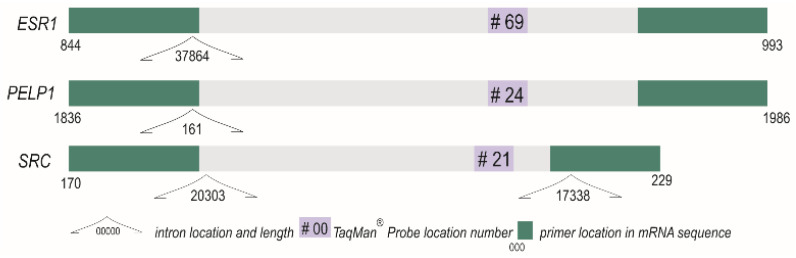
Amplicons of the analyzed genes *ESR1, PELP1* and *SRC* with their primers, TaqMan^®^ Probes and introns positions. *ESR2* and *HPRT* (reference) probe assays are protected by trade secrets.

RNA expression pattern analysis was performed using the LightCycler^®^ 2.0 carousel glass capillary-based system (Roche, Manheim, Germany). Primer sequences and TaqMan^®^ hydrolysis probe position for the gene of interest (GOI) were determined using Roche Universal Probe Library (UPL) Assay Design Center (http://qpcr.probefinder.com, last accessed on 28 September 2017) for *ESR1*, *PELP1* and *SRC.* In the case of *ESR2*, a ready to use assay was purchased (PrimePCR, qHsaCEP0052206, BioRad, Hercules, CA, USA). The hypoxanthine-guanine phosphoribosyltransferase (*HPRT*) gene assay (Roche, Manheim, Germany) served as an internal control. The description and location of probes and primers for the self-designed assays are shown in Table 2 and Figure 8.

The quantitative polymerase chain reactions, with standard cycling and acquisition steps, were conducted as described previously in a total volume of 20 μL [41]. The reaction mixture with Roche UPL probes for *ESR1*, *PELP1,* and *SRC* has been standardized [41]. In case of *ESR2* and *HPRT,* 1× LightCycler^®^ FastStart TaqMan^®^ Probe Master mix (Roche, Manheim, Germany) was used according to the manufacturer’s protocol. Each reaction was performed in duplicate on independently synthetized cDNA, and the mean values were used for statistical analyses.

Reaction efficiencies were obtained from each gene’s own standard curve [41]. Threshold values were analyzed by comparison to appropriately selected standard curves and reference gene assays with the use of LC 5.0.0.38 software (Roche, Manheim, Germany) and presented as a concentration ratio (Cr).

### 4.4. Protein Expression

#### 4.4.1. Western Blot

Tissue specimens were first lysed in RIPA Lysis Buffer (Merck Millipore, Darmstadt, Germany). Total extracts were shaken for one hour at 4 °C and centrifuged (14,000× *g*, 20 min, 4 °C). Protein concentration was determined with QuickStart Bradford 1× Dye Reagent (BioRad, Hercules, CA, USA) and measured colorimetrically (NanoPhotometer^®^ NP80; IMPLEN, München, Germany). Then, 20 µg of protein lysate per lane was diluted with Laemmli buffer (BioRad, Hercules, CA, USA), denaturated (70 °C, 10 min), and loaded onto a 10% SDS-polyacrylamide gel (TGX FastCast Acrylamide Kit 10%, BioRad, Hercules, CA, USA). Electrophoretically separated proteins were transferred to Immobilon PVDF membranes (Merck Millipore, Darmstadt, Germany). Membranes were incubated at room temperature for one hour in blocking buffer (TBS-T with 5% bovine serum albumin) in order to block nonspecific antigen binding. Membranes were then incubated overnight at 4 °C with primary antibodies, diluted in TBS-T buffer with 3% non-fat milk, anti-ESR1 (1:1000, LS-C88420, Lifespan Biosciences, Seattle, WA, USA), ESR2 (1:1000, ab3576, Abcam, Cambridge, UK), PELP1/MNAR (1:1000, A300-180A, Bethyl, Montgomery, TX, USA), SRC (1:1000 orb379229, Biorbyt, Cambridge, UK), p-SRC (1:500, orb14869, Biorbyt, Cambridge, UK), and GAPDH (1:2500, sc-25778, Santa Cruz Biotechnology, Dallas, TX, USA). After incubation with primary antibodies, membranes were washed three times with TBS-T buffer and incubated on an orbital shaker (1 h, RT, 250 rpm) with secondary polyclonal goat anti-rabbit antibody (horseradish peroxidase conjugated, 1:1000, P0448, Dako, Glostrup, Denmark). Blots were visualized by enhanced chemiluminescence using Clarity Western ECL (BioRad, Hercules, CA, USA) and documented with G:BOX (Syngene, Cambridge, UK). The 3-Colour Prestained Protein Marker (Blirt, Gdansk, Poland) was used as the mass standard.

#### 4.4.2. Immunohistochemistry

The protein localization in tissues was assessed using immunohistochemistry as described [42]. In short, formalin fixed, paraffin-embedded tissue specimens were cut in 3 µm thick sections. The slides were boiled in a microwave oven twice (2 × 10 min, 200 W) in citrate buffer (pH 6.0, 0.1 mM citric acid solution, 0.1 mM sodium citrate solution; Avantor Performance Materials Poland S.A., Gliwice, Poland) and then cooled at RT. In order to block endogenous peroxidase activity, slides were incubated for 3 min in 3% hydroxyperoxide solution (Avantor Performance Materials Poland S.A., Gliwice, Poland). To avoid nonspecific antibody binding, the slides were placed in TBS-T buffer (containing 3% BSA) for 1 h at RT. IHC reactions were performed with the use of the previously described primary antibodies at a 1:100 dilution. Reactions were visualized through subsequent incubation with secondary biotinylated goat anti-rabbit immunoglobulins and 3,3′-diaminobenzidine chromogen (K3468, Dako, Glostrup, Denmark). The slides were counterstained with hematoxylin. The assessment was performed using light microscopy via the Olympus CX41 microscope (Olympus Corporation, Tokyo, Japan).

### 4.5. Statistical Analyses

Statistical analyses were performed using Statistica^®^ Version 13.5.0 software for Windows (TIBCO Software Inc., Palo Alto, CA, USA). The obtained results were compared in groups: (1st subgrouping) control (C) vs. ovarian cancer patients (OCP) and (2nd subgrouping) ovary tissue without changes (OWC) vs. noncancerous benign ovarian changes (BOC) vs. ovarian cancer patients (OCP) as shown in the Figure 1. The expression levels were normalized using min-max normalization. To describe experimental results, the median (interquartile range) was used. The Shapiro–Wilk test was used for the normality of continuous variable distribution assessment. The Mann–Whitney U and Kruskal–Wallis tests were used followed by Dunn’s post-hoc tests. The Bonferroni correction was used for multiple comparisons. Correlation coefficient (R) values between parameters were determined using Spearman rank tests. Two-tailed tests for correlation coefficients were used to establish the gene expression and their quotient relationships. Data were considered statistically significant at *p* < 0.05.

## 5. Conclusions

The expression, expression ratios and correlations as well as their mutual interactions in ovarian cancer represent a potentially interesting target in ovarian cancer biology. Some of the gene-to-gene mRNA expression levels interactions are changed in benign malignancies and aberrant in ovarian cancer, especially regarding *PELP1*. The deregulation of estrogen signaling pathway seems to be the key factor associated with ovarian cancer pathology. This study suggests that specific estrogen-mediated functions in the ovary and ovary-derived cancer might result from varying local interactions of estrogens with their receptors and coregulators.

## Figures and Tables

**Table 1:** Characteristics of study groups and results of the qPCR reactions (normalized).

Variables	1st Subgrouping			2nd Subgrouping			
	Controls (*n* = 27) Median (Q1–Q3) ^&^	Ca Ovary (*n* = 25) Median (Q1–Q3) ^&^	*p*-Value ^#^	Ovary without Changes (*n* = 12) Median (Q1–Q3) ^&^	Benign Ovarian Changes (*n* = 15) Median (Q1–Q3) ^&^	Ca Ovary (*n* = 25) Median (Q1–Q3) ^&^	*p*-Value ^@^
Age (years)	53 (45–67)	63 (54.5–69.5)	**0.0445**	58 (49.0–69.0)	51 (44.0–60.0)	63.0 (54.5–69.5)	**0.0404**
BMI	27.14 (22.31–28.96)	26.22 (20.93–30.42)	0.8103	24.84 (22.32–28.67)	27.33 (21.63–31.23)	26.22 (20.93–30.42)	0.9062
Menopause No	N = 11 (85%)	N = 2 (15%)	**0.0068 ^‡^**	N = 4 (31%)	N = 7 (54%)	N = 2 (15%)	**0.0151** ^§^
Menopause Yes	N = 15 (40%)	N = 22 (60%)		N = 7 (19%)	N = 8 (22%)	N = 22 (59%)	
*ESR1* Cr ^$^	0.0042 (0.0002–0.0074)	0.0041 (0.0019–0.0370)	0.2339	0.0042 (0.0006–0.0468)	0.0038 (0.0001–0.0068)	0.0041 (0.0019–0.0369)	0.2579
*ESR2* Cr ^$^	0.0675 (0.0329–0.1193)	0.0329 (0.0157–0.1190)	0.1996	0.0416 (0.0329–0.1106)	0.1020 (0.0675–0.1365)	0.0329 (0.0157–0.1193)	0.0997
*PELP1* Cr ^$^	0.2880 (0.1863–0.5932)	0.1863 (0.0846–0.2880)	0.0602	0.3389 (0.1863–0.5932)	0.2880 (0.0846–0.5932)	0.1863 (0.0846–0.2880)	0.1193
*SRC* Cr ^$^	0.1003 (0.0388–0.1770)	0.0136 (0.0035–0.0340)	**0.0070**	0.1000 (0.0266–0.2077)	0.1003 (0.0379–0.1530)	0.0136 (0.0036–0.0340)	**0.0247**
*ESR1/ESR2*	0.0380 (0.0034–0.1273)	0.1805 (0.0225–0.8400)	0.0842	0.0658 (0.0195–0.4106)	0.0343 (0.0007–0.0653)	0.1805 (0.0225–0.8403)	0.0711
*ESR1/PELP1*	0.0076 (0.0021–0.0183)	0.0598 (0.0100–0.1970)	**0.0189**	0.0076 (0.0034–0.2070)	0.0078 (0.0021–0.0131)	0.0598 (0.0100–0.1969)	**0.0468**
*ESR1/SRC*	0.0282 (0.0103–0.0762)	0.4791 (0.0649–2.1490)	**0.0048**	0.0419 (0.0236–0.7292)	0.0261 (0.0095–0.0604)	0.4791 (0.0649–2.1491)	**0.0079**
*ESR2/PELP1*	0.2156 (0.1720–0.5340)	0.2694 (0.1026–0.5950)	0.9249	0.1744 (0.1240–0.1809)	0.3881 (0.2302–0.7934)	0.2694 (0.1026–0.5953)	0.0842
*ESR2/SRC*	0.7612 (0.4080–3.0752)	3.0844 (0.3503–21.790)	0.2308	0.6691 (0.3079–2.7817)	0.8509 (0.5823–3.0752)	3.0844 (0.3503–21.790)	0.3796
*PELP1/SRC*	3.8765 (1.3271–7.0129)	7.2099 (2.2466–34.277)	0.0808	4.0065 (3.3745–15.903)	2.0548 (0.8433–5.2661)	7.2099 (2.2466–34.277)	0.1167

n number of cases; ^&^ Q1–Q3—lower and upper quartile; ^#^ Mann–Whitney U test; ^@^ Kruskal–Wallis test; **^‡^**V^2^ test; ^§^ maximum likelihood chi-square test; Cr ^$^ concentration ratios data normalized using min-max normalization. Statistically significant differences are indicated in bold. The significant differences in genes’ expression and expression ratios are presented in Figure 1 and Figure 2.

**Table 2:** Quantitative polymerase chain reaction probes and primer designations.

Gene	Manufacturer’s Designation	Cat. No.	Primer Sequence 5’→3’		Amplicon Length (bp)	Manufacturer
*ESR1*	#69	04688686001	F	ccttcttcaagagaagtattcaagg	160	Roche
			R	attcccacttcgtagcatttg		
*ESR2*	dHsaCPE5037392	10041596	*		87	BioRad
*PELP1*	#24	04686985001	F	caaggaggagactcacaggag	131	Roche
			R	caaggaggagactcacaggag		
*SRC*	#21	04686942001	F	gccatgttcactccggttt	100	Roche
			R	cagcgtcctcatctggtttc		
*HPRT*	102079	05532957001	*			Roche

* sequences protected by trade secrets; F—forward primer; R—reverse primer; (bp) base pairs.

## Data Availability

The datasets used and analyzed during the current study are available from the corresponding author on reasonable request.

## Supplementary Materials

The following are available online at https://www.mdpi.com/article/10.3390/ijms22126216/s1.
