# Supplementary material for: Altered Expression of ESR1, ESR2, PELP1 and c-SRC Genes Is Associated with Ovarian Cancer Manifestation
Source: Int J Mol Sci. 2021 Jun 9;22(12):6216. doi: 10.3390/ijms22126216 (PMC8228770; doi:10.3390/ijms22126216)
Supplement: Supplementary file 1 [file ijms-22-06216-s001.zip › ijms-1199991-supplementary.pdf]

Supplementary material

Altered Expression of *ESR1*, *ESR2*, *PELP1* & *SRC* Genes is Associated with Ovarian Cancer Manifestation

Monika Englert-Golon[1&] (ORCID: 0000-0003-1110-8693), Mirosław Andrusiewicz[2&¶] (ORCID: 0000-0002-8781-3447), Aleksandra Żbikowska[2] ORCID: 0000-0002-6891-8569), Małgorzata Chmielewska[2] (ORCID: 0000-0003-0091-6492), Stefan Sajdak[1] (0000-0002-8641-4188), Małgorzata Kotwicka[2] (ORCID: 0000-0002-9802-374X),

[1]Surgical Gynecology Clinic of The Gynecological and Obstetrics Clinical Hospital, Poznan University of Medical Sciences, Polna 33, 60-535 Poznan, Poland.

[2]Chair and Department of Cell Biology, Poznan University of Medical Sciences, Rokietnicka 5D, 60-806 Poznan, Poland

[&] Mirosław Andrusiewicz and Monika Englert-Golon should be considered joint first authors.

Relationship between ER signaling and ovarian cancer

Clinical data correlation

The Spearman rank correlation test showed patient age positively and strongly correlated with BMI in controls (1st subgrouping) and non-cancerous benign ovarian changes (2nd subgrouping). None of the other analyzed clinical variables significantly correlated with age of the patients neither in the cancer patients nor in other distinguished groups (independent of 1st and 2nd subgrouping) (Figure S1).

Gene expression level correlation

Normalized concentration ratio data (referring to relative expression level) correlation results were presented in Figure S1. In case of the 1st subgrouping, in controls, *ESR1* Cr values were proportionally correlated with *PELP1* and *SRC* Cr (weak and moderately, respectively). None of the other correlations were significant in this group, for both gene expression and clinical data. In the ovarian cancer group (1st subgrouping), a moderate correlation was shown with *PELP1* with *ESR1* and *SRC* with both *ESR1* and *PELP1* normalized Crs. Taking into consideration the 2nd subgrouping, in tissues lacking any changes, a strong correlation was observed for BMI and *SRC* Cr as well as *ESR1* and. *ESR2* Cr values. In benign changes of ovary tissue, the normalized Cr values strongly and positively correlated in the case of *SRC* and *PELP1* (Figure S1).

Gene expression quotient ratio correlations

In the 1st subgrouping, the controls, the *ESR1*/*ESR2* ratio correlated proportionally and very strongly with *ESR1* and moderately with *PELP1* Cr. The *ESR1*/*PELP1* ratio correlated positively and very strongly with *ESR1* Cr values and *ESR1*/*ESR2* ratio. Very strong and

proportional correlations were shown in the *ESR1*/*SRC* ratio and *ESR1* Cr and both *ESR1*/*ESR2* and *ESR1*/*PELP1* ratios. In turn, the *ESR2*/*PELP1* ratio was strongly and negatively correlated with *PELP1* Cr. The *ESR2*/*SRC* ratio was moderately and negatively correlated with BMI, *PELP1* Cr values, and weakly correlated with *ESR1*/*ESR2* ratio. A very strong negative correlation was shown with *SRC* Cr. *ESR2*/*SRC* ratio, only in the case of the *ESR2*/*PELP1* ratio, was moderately and positively correlated. In the case of the *PELP1*/*SRC* ratio, negative and strong correlations were shown with BMI and *ESR1*/*ESR2* and positive for the *ESR2*/*SRC* ratio (Figure S1).

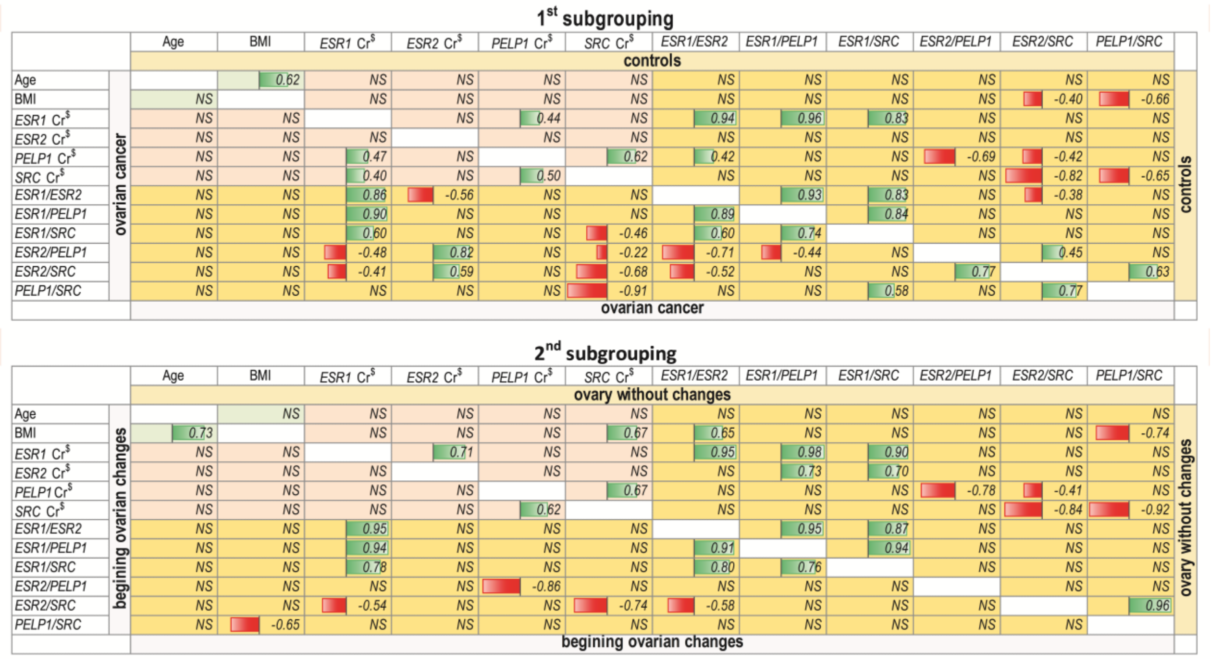

1st subgrouping (upper: controls; lower: ovarian cancer)

	Age	BMI	ESR1 Cr$	ESR2 Cr$	PELP1 Cr$	SRC Cr$	ESR1/ESR2	ESR1/PELP1	ESR1/SRC	ESR2/PELP1	ESR2/SRC	PELP1/SRC
Age		0.62	NS	NS	NS	NS	NS	NS	NS	NS	NS	NS
BMI	NS		NS	NS	NS	NS	NS	NS	NS	NS	-0.40	-0.66
ESR1 Cr$	NS	NS		NS	0.44	NS	0.94	0.96	0.83	NS	NS	NS
ESR2 Cr$	NS	NS	NS		NS	NS	NS	NS	NS	NS	NS	NS
PELP1 Cr$	NS	NS	0.47	NS		0.62	0.42	NS	NS	-0.69	-0.42	NS
SRC Cr$	NS	NS	0.40	NS	0.50		NS	NS	NS	NS	-0.82	-0.65
ESR1/ESR2	NS	NS	0.86	-0.56	NS	NS		0.93	0.83	NS	-0.38	NS
ESR1/PELP1	NS	NS	0.90	NS	NS	NS	0.89		0.84	NS	NS	NS
ESR1/SRC	NS	NS	0.60	NS	NS	-0.46	0.60	0.74		NS	NS	NS
ESR2/PELP1	NS	NS	-0.48	0.82	NS	-0.22	-0.71	-0.44	NS		0.45	NS
ESR2/SRC	NS	NS	-0.41	0.59	NS	-0.68	-0.52	NS	NS	0.77		0.63
PELP1/SRC	NS	NS	NS	NS	NS	-0.91	NS	NS	0.58	NS	0.77	

2nd subgrouping (upper: ovary without changes; lower: begining ovarian changes)

	Age	BMI	ESR1 Cr$	ESR2 Cr$	PELP1 Cr$	SRC Cr$	ESR1/ESR2	ESR1/PELP1	ESR1/SRC	ESR2/PELP1	ESR2/SRC	PELP1/SRC
Age		NS	NS	NS	NS	NS	NS	NS	NS	NS	NS	NS
BMI	0.73		NS	NS	NS	0.67	0.65	NS	NS	NS	NS	-0.74
ESR1 Cr$	NS	NS		0.71	NS	NS	0.95	0.98	0.90	NS	NS	NS
ESR2 Cr$	NS	NS	NS		NS	NS	NS	0.73	0.70	NS	NS	NS
PELP1 Cr$	NS	NS	NS	NS		0.67	NS	NS	NS	-0.78	-0.41	NS
SRC Cr$	NS	NS	NS	NS	0.62		NS	NS	NS	NS	-0.84	-0.92
ESR1/ESR2	NS	NS	0.95	NS	NS	NS		0.95	0.87	NS	NS	NS
ESR1/PELP1	NS	NS	0.94	NS	NS	NS	0.91		0.94	NS	NS	NS
ESR1/SRC	NS	NS	0.78	NS	NS	NS	0.80	0.76		NS	NS	NS
ESR2/PELP1	NS	NS	NS	NS	-0.86	NS	NS	NS	NS		NS	NS
ESR2/SRC	NS	NS	-0.54	NS	NS	-0.74	-0.58	NS	NS	NS		0.96
PELP1/SRC	NS	-0.65	NS	NS	NS	NS	NS	NS	NS	NS	NS	

Figure S1. Correlation coefficient plot of 1st subgrouping (controls and ovarian cancer patients), and 2nd subgrouping (ovarian tissue samples lack of any changes patients and females with beginning non-cancerous changes in the ovary). Designation: *NS* – not significant; numbers – Spearman rank correlation coefficients; green bars – proportional correlation; red bars – reverse proportional correlation. Green background – clinical data correlations; pink background – genes' expression correlations; yellow background – genes expression quotient ratio correlations.

In ovarian cancer patients of the 1st subgrouping, a moderate and negative correlation was shown for the *ESR1*/*SRC* ratio, *ESR2*/*SRC* and *ESR2*/*PELP1 vs. ESR1* Cr values. Both *ESR1*/*ESR2* and *ESR1*/*PELP1* strongly and positively correlated with *ESR1* Cr while *ESR1*/*SRC* moderately correlated with *ESR1* Cr. The *ESR2* Cr values were negatively correlated at the moderate level with the *ESR1*/*ESR2* ratio and positively with the *ESR2*/*SRC* ratio. Strong and positive correlations were observed in case of *ESR2* Cr and the *ESR2*/*PELP1* ratio. None of the ratios correlated significantly with *PELP1* Cr. *SRC* Cr values obtained in the ovarian cancer patients correlated negatively with ratios of *ESR1*/*SRC* (moderately), *ESR2*/*PELP1* (weak), *ESR2*/*SRC* (strong), and *PELP1*/*SRC* ratio (very strong). The correlation of *ESR1*/*ESR2* with

ESR1/*PELP1* and *ESR1*/*SRC* ratios was proportional and very strong. In turn, *ESR1*/*ESR2* was strongly and moderately while being negatively correlated with *ESR2*/*PELP1* and *ESR2*/*SRC* ratios, respectively. Between *ESR1*/*PELP1 vs. ESR1*/*SRC* ratios, strong positive correlations were shown and were negative moderate with *ESR2*/*PELP1* ratios. The *ESR1*/*SRC* ratio was moderately strong and positively correlated with *PELP1*/*SRC*. In case of both, *ESR2*/*PELP1 vs. ESR2*/*SRC* and ESR2/SRC *vs. PELP1*/*SRC*, a positive Spearman rank correlation was found (Figure S1).

In the ovarian tissue without changes of the 2nd subgrouping, the *ESR1*/*ESR2* ratio was positively strong and very strongly correlated with BMI and *ESR1* Cr, respectively. Both ratios, *ESR1*/*PELP1* and *ESR1/SRC*, correlated positively very strong with *ESR1* Cr and *ESR1/ESR2* ratios and strongly with *ESR2* Cr. Additionally, *ESR1/SRC* was very strong positively correlated with the *ESR1*/*PELP1* ratio. The *ESR2/PELP1* ratio was moderately, reverse proportionally correlated with *PEPL1* Cr. A negative correlation was also observed between *ESR2*/*SRC vs. PELP1* Cr (moderately strong), and *SRC* Cr (very strong). The *PELP1*/*SRC* ratio correlated negatively and very strong with *SRC* Cr but positively and very strong with *ESR2*/*SRC* ratio (Figure S1).

In the ovarian tissue with benign, non-cancerous changes of the 2nd subgrouping, *ESR1*/*ESR2* and *ESR1*/*PELP1* ratios both correlated positively very strong and *ESR1*/*SRC* was observed at a strong level with *ESR1* Cr. In turn, *ESR2*/*SRC* correlated negatively and moderately strong with *ESR1* Cr. *ESR2*/*PELP1* correlated negatively very strong with *PELP1* Cr, and *ESR2*/*SRC* was negatively strong with *SRC* Cr and moderately, negatively strong with the *ESR1*/*ESR2* ratio. *ESR1*/*PELP1* and *ESR1*/*SRC* ratios positively and very strong correlated with *ESR1*/*ESR2* ratios. No other correlations were statistically significant in this group (Figure S1).
